# Diagnostic anténatal de la coupole diaphragmatique comprendre et accompagner: facteurs pronostique

**DOI:** 10.11604/pamj.2021.39.9.28895

**Published:** 2021-05-03

**Authors:** Imane Attar, Hekmat Chaara, Sofi Jayi, Fatima-Zahra Fdili Alaoui, Moulay Abdelilah Melhouf

**Affiliations:** 1 Service de Gynécologie et d´Obstétrique II, CHU Hassan II, Fès, Maroc

**Keywords:** Hernie de la coupole diaphragmatique fœtale, diagnostic anténatal, *lung over head ratio*, facteur pronostic, Congenital diaphragmatic hernia, antenatal diagnosis, lung over head ratio, prognostic factor

## Abstract

Nous avons mené une étude portée sur cinq cas d´hernie de la coupole diaphragmatique fœtale (HCD) sur deux ans au sein de notre unité de diagnostic anténatal afin de faire un rappel sur les repères généraux concernant cette pathologie et sa prise en charge toute en soulignant les actualités en matière d'évaluation prénatale du pronostic néonatale à l´aide du calcul du Lung over Head Ratio (LHR): échographie versus imagerie par résonance magnétique (IRM) dans les pays du tiers-monde. Le but de notre travail est de clarifier le devenir de ces nouveau-nés et d'assurer un accompagnement au couple d'un fœtus portant une hernie de la coupole diaphragmatique.

## Abstract (en)

We conducted a study of five patients with congenital diaphragmatic hernia (CDH) in the department of antenatal diagnosis over a period of two years. Our study highlights the overall benchmarks for this disease and its management as well as the news on prenatal assessment of neonatal prognosis using Lung Over Head Ratio (LHR): ultrasound versus magnetic resonance imaging (MRI) in the third world. The purpose of this study is to evaluate the prognosis of these new-born babies and support parents of a fetus with diaphragmatic hernia.

## Introduction

La HCD se définit par l´issue des viscères abdominaux dans le thorax à travers un orifice diaphragmatique malformatif, responsable d´une hypoplasie pulmonaire et d´anomalies structurelles et fonctionnelles de la circulation pulmonaire [1]. Elle est plus fréquente à gauche (90% des cas) qu´à droite (10% des cas) et elle reste exceptionnellement bilatérale [2,3]. Relativement fréquente avec une incidence de 1/2000 à 1/5000 naissances, représentant près de 8% de l´ensemble des malformations congénitales majeures. Elle peut être associée à d´autres malformations et/ou des anomalies chromosomiques (cardiaques, digestives, syndrome de Fryns, trisomie 18 ou 13) [2,4]. Malgré les énormes progrès réalisés dans la compréhension de la physiopathologie de la HDC, l´étiologie et la pathogénie relatives à cette malformation ne sont pas encore complètement élucidés [8], offrant l'opportunité d'être référés vers un centre de soins tertiaires pour une prise en charge périnatale. Dans des cas isolés, l'issue peut être prédite avant la naissance par imagerie médicale. La combinaison de la taille du poumon et de la hernie hépatique est une méthode largement acceptée pour stratifier les fœtus en groupes présentant un degré croissant d'hypoplasie pulmonaire et des taux de mortalité correspondants [9]. Ici, nous allons essayer d´appliquer les dernières recommandations du protocole d'évaluation radiologique (échographie et IRM) standardisée sur les fœtus avec HCD isolée et la prédiction individualisée de l'issue néonatale.

## Méthodes

Nous avons mené une étude prospective non randomisée, monocentrique observationnelle chez des fœtus diagnostiqués porteurs d´HDC au service de gynécologie obstétrique du CHU Hassan II de Fès entre septembre 2018 et décembre 2020. On a inclus tous les fœtus atteints de HCD diagnostiqués en anténatale. Les critères d´exclusion étaient les cas non isolés dans le cadre d´un syndrome polymalformatif. Les paramètres étudiés étaient les suivants: âge maternel et paternel, antécédent (ATCD) de malformations, âge gestationnel de diagnostic, topographie, la position d´estomac et du foie, LHR en échographie et volume pulmonaire en IRM, terme et voie d´accouchement, APGAR, le poids à la naissance ainsi que les mesures de réanimations néonatales. Tous les examens ont été réalisés par voie trans abdominale, avec un échographe Voluson 730 Expert. Pour la mesure de la zone pulmonaire, une section transversale de la poitrine du fœtus contenant la vue à quatre chambres du cœur et les zones de chaque poumon ont été mesurées dans ce plan échographique en premier par un tracé manuel des limites des poumons; Deuxièmement en multipliant le diamètre le plus long du poumon par son diamètre perpendiculaire le plus long. En outre, la circonférence de la tête a été mesurée à l´aide du diamètre bipariétal standard, montrant l'écho de la ligne médiane divisant le cerveau en deux hémisphères égaux, le cavum de septum pellucidum et les cornes postérieures des ventricules latéraux. Ensuite le LHR a été calculé en divisant la surface de chaque poumon (mm^2^) par le tour de la tête (mm). Les valeurs LHR o/a ont ensuite été calculées en entrant ces mesures dans le calculateur LHR sur perinatology.com. Un seul examinateur a analysé les images échographiques pour déterminer la précision des calculs. Les IRM ont également été revues par un seul radiologue pédiatrique. Les patientes ont été suivies en consultation mensuelle jusqu´au terme. Au cours de chaque consultation on réalisait une mesure de la LHR, revérifier le contenu du sac herniaire toute en s´assurant du bien-être fœtal. Le but principal et d'évaluer le pronostic en étudiant deux principaux paramètres: la mesure de LHR, et la position du foie; alors que le but secondaire était de comparer les résultats obtenus en échographie avec ceux de l´IRM.

## Résultats

Cinq cas d´HCD isolé ont été collectées, Le sexe-ratio est de 1,03, L´âge maternel moyen est de 27,8ans, l´âge paternel moyen de 36,8ans, La récurrence intrafamiliale est nulle, La fécondation est naturelle chez toutes nos patientes , l´âge gestationnelle moyenne de diagnostic étais de 26,4SA, l´âge moyenne d´accouchement étais de 35,8SA, la voie d´accouchement étais par césarienne programmée pour 2 patientes et 2 ont arrivée à dilatation complète à la salle d´accouchement et une voie basse a été accepté alors que le 5e cas a été déclenché et livré par voie vaginale suite à un MFIU, Le poids moyen de naissance (PMN)est de 2600 grammes, l´APGAR moyen était de 6,6/10 (Tableau 1). Toutes les patientes ont bénéficiers d´une analyse morphologique détaillée ainsi qu´une étude génétique revenants normale ce qui a confirmé le caractère isolé de nos 5 cas, la hernie était du côté gauche dans 4 cas et bilatérale au 5e cas. La recherche échographique du contenu de sac herniaire s´est focalisée essentiellement sur la localisation du foie suspecté en intrathoracique chez un seul cas mais repositionner en intra-abdominale après complément IRM. La moyenne de LHR calculé par échographie était de 1,2 versus une moyenne de 1 sur l´IRM, alors que la moyenne LHR o/a par échographie et IRM était respectivement de 36% et 36,6% (Tableau 2).

**Tableau 1:** caractéristiques épidémiologiques de la population étudiée

	Age maternel (ans)	Age paternel (ans)	Age gestationnelle (SA)	Le Terme (SA)	La Voie d´accouchement	Le PMN (gr)	Apgar
Cas n°1	35	40	28	39	VH	3200	9/10
Cas n°2	27	38	32	37	VB	2800	8/10
Cas n°3	28	39	22	28	VB	700	0/10
Cas n°4	25	35	23	39	VH	3400	9/10
Cas n°5	24	32	27	36	VB	2900	7/10
La moyenne	27,8	36,8	26,4	35,8	-	2600	6/6
L´écart type	4,3	3,2	4,03	4,5	-	1088,5	3/7

**Tableau 2:** résultats échographique et IRM pour le calcul de la LHR et de la LHR o/a ainsi que le contenu de sac herniaire

	Echographie				IRM fœtal			
	LHR	LHR o/a	Position du Foie	Position estomac	LHR	LHR o/a	Position du Foie	Position estomac
Cas n°1	1,2	35%	Abdominale	Thoracique	1,3	30%	Abdominale	Thoracique
Cas n°2	1,53	67%	Abdominale	Abdominale	1,29	55%	Abdominale	Abdominale
Cas n°3	0,72	13 %	Thoracique	Thoracique	1	16%	Abdominale	Thoracique
Cas n°4	1,6	55%	Abdominale	Thoracique	1,45	45%	Abdominale	Thoracique
Cas n°5	0,4	11%	Abdominale	Thoracique			-	-
Moyenne	1,2	36%	-	-	1	36,6%	-	-

Les 2 nouveau-nés délivrés par césarienne programmer avec un pronostic estimé à 95% (cas N°2, cas N°4) on était hospitalisé au service de réanimation néonatale après une intubation immédiate à la salle d´accouchement puis décédé à J1 de vie dans un contexte d´arrêt cardio respiratoire; les 2 autres accouchées par voie basse (cas N°1=taux de survie 40-60%, cas N°3=taux de survie inf. à 5%) ont décédé une heure de vie. Quoique que toutes nos patientes on bénéficiait des dernières recommandations en matière d´évaluation anténatale du pronostic post natale basé sur la mesure de la LHR par échographie et IRM avec une correspondance jugée optimale. Tous les bébés sont morts chose qui peut être expliquée par la non-disponibilité d´un plateau technique dédié à accueillir ce genre de malformation assez délicate et qui nécessite un certain niveau d´expérience. Cette expérience à faible échantillon nous a permis quand même de traquer le niveau où il y a une défaillance lors de la prise en charge de cette anomalie jugée curable, nous recommandant ainsi de réserver une certaine attention pour améliorer les structures sanitaires accueillant ces bébés.

## Discussion

**Figure 1:**
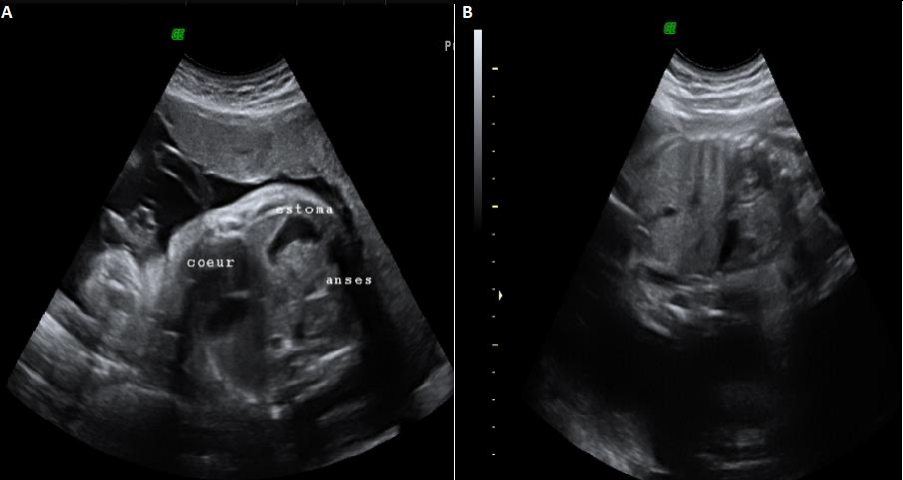
image échographique en coupe transversal (A) et en coupe sagittale montrant l´ascension de l´estomac et des anses en intrathoracique

**Figure 2:**
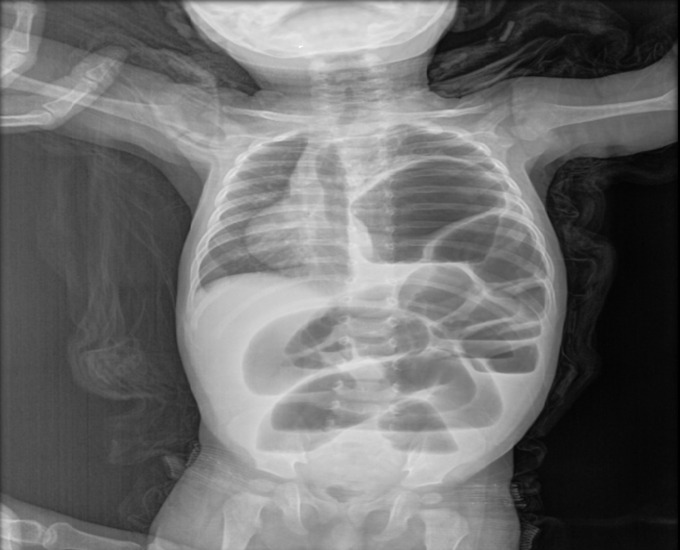
radiographie standard H10 de vie: présence de clartés digestive en intra thoracique gauche avec importante distension colique d´amont, il s´y associe un refoulement du médiastin vers le coté controlatéral

**Critères diagnostique de la HCD:** au cours des 20 dernières années, les taux de détection prénatale de l´HCD se sont considérablement améliorés, passant de 15% au milieu des années 80 à près de 60% à la fin des années 90; dans la grande majorité des cas, l'anomalie est détectée lors de l´échographie morphologique du 2^e^ trimestre de routine, avec un âge moyen de 22 à 24 semaines, dans certains cas, la HCD peut être diagnostiquée même au cours du premier trimestre, cependant Près de 11% des cas sont manqués pendant la période prénatale et diagnostiqués après la naissance; dans notre série l´âge moyen de diagnostic était de 26SA [10]. i) HCD gauche: le diagnostic repose principalement sur la visualisation des organes abdominaux en intrathoracique avec un déplacement médiastinal vers la droite, causé par une hernie de l'estomac vue sur la coupe des quatre cavités. Cette dernière ainsi que l´intestin grêle prennent l´aspect d´images liquidiennes animées d´un mouvement péristaltique qui se distinguent facilement du poumon fœtal plus échogène (Figure 1, Figure 2). La visualisation directe du défet diaphragmatique est très difficile étant donné que seule une partie du diaphragme peut être défectueuse. En absence des signes pathognomonique, la recherche des signes indirectes est d´une utilité diagnostique importante et cela comprennent: ii) la déviation ainsi qu´une compression du médiastin du côté opposé à la hernie ce qui peut provoquer des hydrops fœtaux. iii) L´échogénicité anormale de l´hémithorax. iv) L´hydramnios, par compression œsophagienne en gênant la déglutition normale ou en créant une obstruction gastrique. v) Le retard de croissance intra-utérin et la diminution du diamètre abdominal transverse. vi) Anomalie de courbure de l´aorte descendante sur une coupe sagittale thoracique: le rachis et l´aorte ne sont plus parallèles [11].

**Figure 3:**
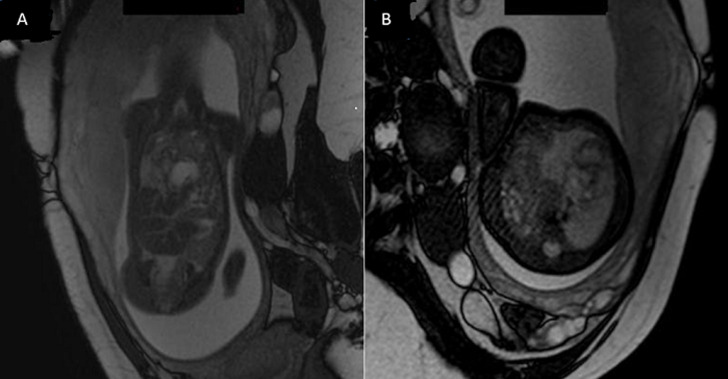
images fœtale par résonnance magnétique en coupe coronal (A) et axial (B) d´une hernie de la coupole diaphragmatique gauche

HCD droite sont moins fréquentes, ils sont plus difficiles à détecter, en raison de la grande similitude d´échogénicité entre le poumon et le foie qui est le plus souvent le seul organe hernié. L'interrogation Doppler de la veine ombilicale et des vaisseaux hépatiques, ou l'emplacement de la vésicule biliaire peuvent être utilisés comme repères supplémentaires pour définir la position du foie. Il faut savoir que la hernie hépatique n´est pas l´apanage des HCD droit mais elle peut s´observer même dans la forme gauche [11]. L'IRM fœtale est largement utilisée dans l'évaluation des troubles pulmonaires fœtales, y compris la HCD (Figure 3). Contrairement à l'échographie, il n'est pas limité par l'obésité maternelle ou l'oligohydramnios, elle offre un meilleur contraste des tissus mous et permet de détecter la présence d'anomalies supplémentaires. Cependant, en raison de son manque de disponibilité dans les pays en voie de développement, l'échographie reste l´examen de référence sur laquelle reposent les décisions concernant la prise en charge prénatale [12]. Dans notre étude les résultats de l´IRM fœtale n´ont pas ajouté des informations supplémentaires en matière de diagnostic. Avant de retenir le diagnostic il est indispensable d´éliminer certains diagnostics différentiels [13]: i) une masse échogène intra thoracique: séquestration, Une forme microkystique de malformation adénomatoïde pulmonaire ou une masse médiastinale (tératome, lymphangiome kystique). ii) Une structure liquidienne dans le thorax: hydrothorax localisé, malformation adénomatoïde pulmonaire macro kystique, lymphangiectasie. iii) Les malformations broncho-œsophagiennes: kyste bronchogénique et neuroentérique, la duplication digestive et l´atrésie bronchique. iv) Une tumeur solide (Tumeur cardiaque, Rhabdomyosarcome, Leiomyosarcome, tumeur neurogène). v) Les anomalies de position du cœur: le situs inversus, Le syndrome d´Ivemark, une hypoplasie cardiaque gauche.

**Critères pronostiques de l´HCD:** une fois le diagnostic est établi, la confirmation de son caractère isolé par une analyse morphologique détaillé et la réalisation d´un caryotype est obligatoire, toute anomalie associée va aggraver le pronostic en dehors des autres facteurs pronostic et une interruption de grossesse peut être discutée avec le couple. Notre discussion va se focaliser sur l´étude des facteurs pronostic d´HCD isolé.

**Figure 4:**
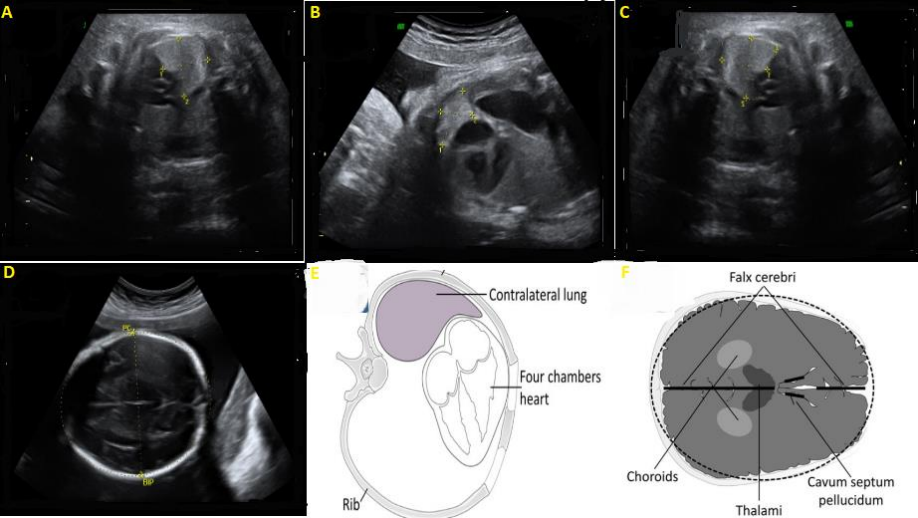
images échographiques des sections pour le calcul du LHR (A-B: méthode de diamètre le plus long sur le diamètre transverse; C): méthode de traçage) sur la vue à 4 chambres; D) plan standard pour la mesure du périmètre crânien; dessins schématiques (E-F)

**Volume pulmonaire:** parce que l'hypoplasie pulmonaire est le principal prédicteur de la mortalité et de la morbidité, il était logique d'essayer d'évaluer le volume pulmonaire avant la naissance par ultrasons. Mektus *et al*. ont d'abord décrit l'utilisation du LHR, fournissant ainsi une évaluation indirecte du volume pulmonaire controlatéral et donc la probabilité d'hypoplasie pulmonaire. Le LHR était considéré comme un marqueur pronostique indépendant de l'âge gestationnel. Néanmoins, il a été démontré plus tard que les deux poumons se développaient plus rapidement que la circonférence de la tête et que LHR augmentent avec l'âge gestationnel. Donc il est inapproprié d'utiliser ce marqueur dans une large fourchette de gestation, c´est pour cela le LHR observé à attendue (O/E) a été introduit comme une mesure indépendante à l'âge gestationnel au moment de la mesure [9]. Il y a un manque de consensus concernant la technique et l'efficacité de la mesure du volume pulmonaire; un éditorial récent de Jani, Peralta et Nicolaides a souligné la nécessité de standardiser la technique de mesure mais jusqu´à nos jours la méthode adopter se résume comme suit (Figure 4): i) le poumon controlatéral au CDH est mesuré dans un plan axial au niveau de la vue à 4 chambres du cœur. Pour obtenir la mesure la plus précise, le poumon est idéalement proche du transducteur à ultrasons. ii) Pour éviter les ombres, le transducteur doit être parallèle aux côtés du fœtus et, par conséquent, un maximum de 2 côtes doit être visible. iii) La zone pulmonaire controlatérale est mesurée à l'aide de la méthode de traçage, qui permet une estimation précise de la zone même si la forme du poumon est irrégulière. En outre, la mesure de diamètre le plus long sur le diamètre transverse et aussi pratique. iv) Cela fournit une zone pulmonaire en millimètres carrés. Il est ensuite divisé par la circonférence de la tête (en millimètres) pour obtenir le LHR. v) Pour obtenir la LHR o/e, la LHR mesurée (= observée) ci-dessus est divisée par la LHR moyenne attendue chez un fœtus normal de AG similaire. Le LHR attendu peut-être obtenu sur un calculateur automatisé sur perinatology.com.

Mais malheureusement la mesure du LHR et LHR O/E ne permet pas de prédire l'état du lit vasculaire pulmonaire et les éventuels échanges gazeux au niveau des alvéoles. Les mesures des branches pulmonaires et les études Doppler du flux vasculaire pulmonaire et de la réactivité n'ont pas encore trouvé de place dans l'évaluation de routine des HCD et dans la prédiction de l'hypertension pulmonaire. De ce fait, l´estimation prénatale du volume pulmonaire reste la seule méthode qui permet de prédire un Pronostic individualisé pour chaque fœtus en fonction de la LHR et la LHR O/E mesuré: vi) si LHR est égal ou inférieur à 1 le pronostic est mauvais, si LHR est entre 1 et 1,4 une oxygénation membranaire extracorporelle (ECMO) est souvent nécessaire et si LHR est plus que 1,4 le pronostic est bon. vii) La survie est proche de 100% avec LHR O/E sup 45%; de 50% avec LHR O/E entre 25-45% et médiocre avec LHR O/E inf 25%. Comme pour le diagnostic, l´IRM fœtale est actuellement la technique de référence pour l'estimation du volume pulmonaire fœtal. L´imagerie par résonance magnétique fournit une mesure plus fiable des poumons controlatérale et même ipsilatéral. La formule la plus utilisée pour le volume pulmonaire total observé à attendu est celle rapportée par Gorincour *et al*. Des données publiées antérieurement suggèrent que l'IRM O/E ≤25% est associée à une mortalité de 100%, et l'IRM O/E ≥46% est associée à un taux de survie de près de 90%. Au cours de ce travail on a essayé de comparer les résultats obtenus par IRM à ceux de l´échographie et on a conclu que l´écart observé n´était pas significatif et que le pronostic présumé était le même en échographie qu´en IRM. De ce fait devant une difficulté a réalisé une IRM l´évaluation échographique semble être suffisante. Cela doit être confirmé par une comparaissant avec un grand nombre d´échantillon.

**Position du foie:** la position échographique du foie est généralement exprimée sous la forme d'une variable binaire non quantitatif « bas » (intra-abdominale) ou « haut » (intrathoracique): ascensionné à différents degrés allant de la simple ascension d´une partie du lobe gauche à une luxation de la majeure partie du foie dans le thorax) et cela est associées à des taux de mortalité de 57% et 7% respectivement. Mais est limité par la grande similitude d´échogénicité entre le foie et le poumon. Cannie *et al*. ont récemment publié une mesure permettant d´évaluer l´ascension du foie en fonction de la position de l´estomac qui semble être prometteuse et devrait se généraliser [14]. Une fois de plus l´IRM permet une analyse plus échelonnée qu´en échographie avec une prédiction quantitatif de volume hépatique hernié mais Malheureusement, il n'existe pas de méthode standardisée pour ce calcule que ça soit en échographie ou en IRM, de ce fait la précision de positionnement hépatique reste le seul indicateur pour prédire la participation du foie pour évaluer le pronostic fœtal. Dans notre série l´évaluation échographique du positionnement hépatique a douté chez un seul fœtus sur un foie intrathoracique mais un complément IRM à redresser le diagnostic en montrant un foie intraabdominal [14].

**Autres facteurs pronostique:** l'importance de l'âge gestationnel au moment du diagnostic est controversée. Certaines études ont montré qu'un diagnostic avant 25 semaines de gestation est prédictif d'un mauvais pronostic, avec des taux de survie de 0% à 56%, alors qu'un diagnostic posé après 25 semaines de gestation était associé à une survie de 100%. Dans notre série l´âge moyen de diagnostic était de 26 SA, aucune patiente n´a bénéficié d´une échographie au 1^er^ trimestre ou lors de la première moitié de 2^e^ trimestre vue le retard de consultation empêchant ainsi l´étude de ce facteur pronostic. Un autre facteur qui reste aussi un sujet de controverse c´est la latéralité de l´HCD. Plusieurs études on montrer une survie globale plus bas en cas de HCD droite. Mais comme il est rare, il sera très difficile de démontrer avec une puissance suffisante cette conclusion. De plus, la hernie hépatique n'est pas discriminante dans le CDH droit, car elle est presque toujours présente. Dans notre série aucun cas HCD droit n´a été diagnostiqué du coup ce paramètre n´a pas été analysé. Quant à la forme bilatérale le pronostic est reconnu comme étant fatale et cela est confirmé dans notre série vue que le cas avec HCD bilatérale a présenté une mort fœtal in-utéro. La recherche de nouveaux facteurs pronostiques continue. Une étude menée chez 21 fœtus avec HCD (23-33 SA), utilisant le doppler 3D afin de mesurer des index de vascularisation pulmonaire a rapporté que ces index étaient significativement plus bas chez les enfants décédés en postnatal par rapport à ceux qui ont survécu avec une sensibilité de 100%. Cependant, ces mesures ne sont pas réalisables en routine [14,15].

**Prise en charge thérapeutique ante et postnatale:** la prise en charge anténatale consiste surtout à préparer l´accouchement qui doit être programmé dans une structure tertiaire sans aucune recommandation sur le mode de délivrance, toute en évaluant le pronostic fœtal par les méthodes sus cite pour accompagner au mieux les parents, la finalité de ces critères pronostiques doit être expliquée. Mais les parents doivent être également informés de toutes les complications inattendues possibles ainsi que des différentes modalités de décès. C'est en ayant une information éclairée, que les parents pourront décider au mieux le devenir de leur bébé. L´IMG peut être discutée pour les cas dont les chances de survie sont évaluées comme étant inférieures à 15-20%, mais cette indication est débattue, dans notre série aucun interruption médicale de grossesse n´a été réalisée. Évaluer un mauvais pronostic permet également au couple qui ne souhaite pas interrompre une grossesse d´envisager et de se préparer à un accompagnement postnatal. La stratégie thérapeutique actuelle pour favoriser la croissance pulmonaire dans les cas graves consiste en une occlusion trachéale endoluminale fœtoscopie percutanée qui a prouvé son efficacité selon plusieurs séries, mais qui reste une intervention couteuse qui dépasse la capacité de certains pays comme le nôtre [16]. Concernant la prise en charge postnatale, elle est bien codifiée avec des recommandations qui précisent les modalités de ventilation, de la gestion hémodynamique et du traitement de l´HTAP mais qui nécessite à son tour une équipe expérimenté pour accueillir ce type de malformations aussi non disponible dans les pays en voie de développements [17]. Toute cette démarche thérapeutique nécessite une prise en charge dans une structure tertiaire en présence d´un plateau technique bien développé et des personnelles qualifier (obstétricienne, néonatologie et chirurgienne pédiatre).

## Conclusion

Les mères qui portent un fœtus avec CDH devraient recevoir des conseils personnalisés sur le résultat attendu qui fait appel à une imagerie prénatale standardisée et rigoureuse, au moment actuel la taille des poumons et la position du foie sont de bons prédicteurs de survie dans le HCD. La précision échographique pour mesurer ces deux paramètres semble prometteuse entre la main d´un obstétricien expérimenté mais en cas de doute le recours à l´IRM semble nécessaire. Les nouveau-nés doivent être pris en charge par des protocoles standardisés dans des centres spécialisés. La thérapie prénatale dans notre structure reste toujours inaccessible mais nous prévoyons améliorer le plateau technique pour accueillir ses nouveaux nés afin d´améliorer leur pronostic postnatal.

### Etat des connaissances sur le sujet

- Le diagnostic et le conseil prénatals de cette entité reposent sur l’évaluation complète dans un centre tertiaire utilisant des tests génétiques avancés, des méthodes d'imagerie modernes, un pronostic individualisé et une familiarité avec la prise en charge multidisciplinaire pré et postnatale de la CDH;
- Le pronostic des formes isolées s´est amélioré ses dernières décennies.

### Contribution de notre étude à la connaissance

- Au moment actuel la taille des poumons et la position du foie sont de bons prédicteurs de survie dans les HCD isolées;
- La précision échographique pour mesurer ces deux paramètres semble prometteuse entre la main d´un obstétricien expérimenté mais en cas de doute le recours à l´IRM semble nécessaire;
- C´est une pathologie dont le pronostic est toujours sombre dans les pays de tiers monde vue la non-disponibilité d’un centre dédier à accueillir ses bébés.

## References

[1] Witters I, Legius E, Moerman P, Deprest J, Van Schoubroeck D, Timmerman D (2001). Associated malformations and chromosomal anomalies in 42 cases of prenatally diagnosed diaphragmatic hernia. Am J Med Genet.

[2] Butler N, Claireaux AE (1962). Congenital diaphragmatic hernia as a cause of perinatal mortality. Lancet.

[3] Langham MR, Kays DW, Ledbetter DJ, Frentzen B, Sanford LL, Richards DS (1996). Congenital diaphragmatic hernia; epidemiology and outcome. Clin Perinatol.

[4] Sweed Y, Puri P (1993). Congenital diaphragmatic hernia: influence of associated malformations on survival. Arch Dis Child.

[5] Hoboth N (1962). Drugs and congenital abnormalities. Lancet.

[6] Brachen MB, Berg A (1983). Bendictin (Debendax) and congenital diaphragmatic hernia. Lancet.

[7] Pollock LD, Hall JG (1979). Posterolateral (Bochdalek) diaphragmatic hernia in sisters. Am J Dis Child.

[8] Gallot Gallot D, Coste K, Francannet C, Laurichesse H, Boda C, Ughetto S (2006). Antenatal detection and impact on outcome of congenital diaphragmatic hernia: a 12-year experience in Auvergne (France). Eur J Obstet Gynecol Reprod Biol.

[9] Metkus AP, Filly RA, Stringer MD, Harrison MR, Adzick NS (1996). Sonographic predictor of survival in fetal diaphragmatic hernia. J Pediatr Surg.

[10] Dillon E, Renwick M, Wright C (2000). Congenital diaphragmatic herniation: antenatal detection and outcome. Br J Radiol.

[11] Claus F, Sandaite I, Dekoninck P, Oscar M, Rogelio CM, Tim Van M (2011). Prenatal anatomical imaging in fetuses with congenital diaphragmatic hernia. Fetal Diagn Ther. Fetal Diagn Ther.

[12] Jani JC, Cannie M, Peralta CF, Jan AD, Kypros HN, Steven D (2007). Lung volumes in fetuses with congenital diaphragmatic hernia: comparison of 3D US and MR imaging assessments. Radiology.

[13] Graham G, Devine PC (2005). Antenatal diagnosis of congenital diaphragmatic hernia. Semin Perinatol.

[14] Cannie M, Jani J, Chaffiotte C, Vaast P, Deruelle P, Houfflin-Debarge V (2008). Quantification of intrathoracic liver herniation by magnetic resonance imaging and prediction of postnatal survival in fetuses with congenital diaphragmatic hernia. Ultrasound Obstet Gynecol.

[15] Coakley FV, Lopoo JB, Lu Y, Hricak H, Albanese CT, Harrison MR (2000). Normal and hypoplastic fetal lungs: volumetric assessment with prenatal single-shot rapid acquisition with relaxation enhancement MR imaging. Radiology.

[16] DiFiore JW, Fauza DO, Slavin R, Peters CA, Fackler JC, Wilson JM (1994). Experimental fetal tracheal ligation reverses the structural and physiological effects of pulmonary hypoplasia in congenital diaphragmatic hernia. J Pediatr Surg.

[17] Storme L, Rakza T, Sfeir R, Aubry E, Pennaforte T, Bonnevalle M (2009). Pour le Centre de Référence maladies rares: hernie de coupole diaphragmatique. Prise en charge médicale per et postnatale de la hernie congénitale du diaphragmatique. Revue de Médecine Périnatale.

